# An Importin-β-like Protein from *Nicotiana benthamiana* Interacts with the RNA Silencing Suppressor P1b of the Cucumber Vein Yellowing Virus, Modulating Its Activity

**DOI:** 10.3390/v13122406

**Published:** 2021-11-30

**Authors:** Beatriz García, Leonor Bedoya, Juan Antonio García, Bernardo Rodamilans

**Affiliations:** Centro Nacional de Biotecnología CNB, Consejo Superior de Investigaciones Científicas CSIC, 28049 Madrid, Spain; bggarcia@cnb.csic.es (B.G.); leonorbedoya@gmail.com (L.B.); jagarcia@cnb.csic.es (J.A.G.)

**Keywords:** *Potyviridae*, RNA silencing suppression, importin β, P1b, cucumber vein yellowing virus

## Abstract

During a plant viral infection, host–pathogen interactions are critical for successful replication and propagation of the virus through the plant. RNA silencing suppressors (RSSs) are key players of this interplay, and they often interact with different host proteins, developing multiple functions. In the *Potyviridae* family, viruses produce two main RSSs, HCPro and type B P1 proteins. We focused our efforts on the less known P1b of cucumber vein yellowing virus (CVYV), a type B P1 protein, to try to identify possible factors that could play a relevant role during viral infection. We used a chimeric expression system based on plum pox virus (PPV) encoding a tagged CVYV P1b in place of the canonical HCPro. We used that tag to purify P1b in *Nicotiana-benthamiana*-infected plants and identified by mass spectrometry an importin-β-like protein similar to importin 7 of *Arabidopsis thaliana*. We further confirmed the interaction by bimolecular fluorescence complementation assays and defined its nuclear localization in the cell. Further analyses showed a possible role of this *N. benthamiana* homolog of Importin 7 as a modulator of the RNA silencing suppression activity of P1b.

## 1. Introduction

Plant viral infections are based on a myriad of protein interactions that represent the fight between the host, trying to stop pathogen replication and spread, and the virus, searching to exploit plant resources for its own benefit [1,2,3]. Some interactions are pro-viral, and some are pro-plant, and in some cases, the same protein could be essential for plant defense and, at the same time, be important during the viral life cycle [4,5]. This complexity reflects the elaborate interplay developed during the plant–virus co-evolution process [6].

One of the main assets viruses use against plant defenses, specifically RNA silencing, are RNA silencing suppressors (RSSs), and practically all plant viruses express at least one protein with this specific function [7,8,9]. *Potyviridae*, the largest family of plant RNA viruses, is no exception to this. Most of its members are monopartite, presenting a single-stranded positive sense RNA of ~10 Kb [10,11,12], whose main ORF encodes a large polyprotein that is processed by viral endopeptidases [13,14]. A secondary ORF, produced after polymerase slippage [15,16,17], encodes an extra product essential for viral movement [18,19]. Apart from the secondary role as RSS of the VPg of some potyviruses [20,21] and the exceptionality of the enlarged P1 proteins of sweet potato infecting potyviruses [8,17,22,23], there are two proteins in the family that function as RSS: HCPro and P1, specifically type B P1. HCPro is a well-known multifunctional protease that helps in aphid transmission and viral particle formation, acting as an RSS at least in potyviruses and rymoviruses [24]. It is a protein extensively studied, and the list of identified interacting host factors continuously grows [4,24,25,26]. Type B P1 proteins are less characterized. They take on the role of RSSs in cases in which the virus encodes no HCPro, such as the ipomovirus known as cassava brown streak virus [27], or when HCPro does not have RSS activity, as is the case of tritimoviruses [28] and poaceviruses [29].

Cucumber vein yellowing virus (CVYV) is an ipomovirus that infects *cucurbitaceae* species, such as cucumber, melon, and watermelon [30]. It does not encode HCPro and presents at the beginning of the polyprotein two P1 endopeptidases of type A and type B (named P1a and P1b, respectively) [31]. P1a is involved in host range definition [32,33], while P1b is the main viral RSS [34]. This protease bears the LX_2_AX_6_ + Zn finger motif characteristic of type B P1 proteins [22,35] and functions as an RSS by sequestering small RNAs, resembling HCPro [36] and probably other type B P1 proteases in the family [37,38,39]. P1b host partners are basically unknown, and only recently the host factor HYD1 was described as a possible interacting protein playing a defensive role during viral infection [40].

Finding plant interactors of this key viral protein would increase our knowledge about potyviral infections and could provide new tools to develop novel antiviral strategies. Thus, we focused our efforts on identifying new interactors of CVYV P1b in *Nicotiana benthamiana*. We used a chimeric virus based on plum pox virus (PPV), in which PPV HCPro was replaced by CVYV P1b. To facilitate P1b purification during viral infection, we included an N-terminal tag, similar to the one used for the identification of host partners in the case of HCPro of potato virus A (PVA) [41]. We managed to identify as the main candidate an importin-β-like protein, homolog of Importin 7 of *Arabidopsis thaliana*, with multiple roles inside the cell: nuclear transport [42], ABA signaling and response [43], and modulation of the miRNA pathway [44]. Our characterization of the interaction between P1b and the *N. benthamiana* homolog of Importin 7 and its effect in the plant identified a new possible role of this host protein as a regulator of the action of viral RSSs.

## 2. Materials and Methods

### 2.1. Plasmids

Plasmids were generated using standard molecular cloning procedures. PCR reactions were performed with Phusion High-Fidelity DNA Polymerase (New England BioLabs, Ipswich, MA, USA). Primers were synthesized by Merck (Darmstadt, Germany), and sequencing of all products was carried out by Macrogen (Seoul, Korea). The primers are listed in Table S2. Restriction enzymes and T4-DNA ligase were purchased from Thermo Fisher Scientific (Waltham, MA, USA) and New England BioLabs (Ipswich, MA, USA). pPPV has been previously described in [45], and pPPV-SIIIP1b was obtained by modifying the reported pPPV_∆HC_ plasmid [45]. The SIIIP1b fragment was obtained by PCR amplification using primers 2712/2711 and using as a template a previously generated pICPPV-SIIIP1b plasmid (data not shown). The resulting product was ligated to pPPV_∆HC_ digested with *Swa*I, and this enzyme was included in the reaction mix to reduce background colonies [46]. pENTRY-Imp7 cDNA was obtained by reverse transcription with Superscript III (Thermo Fisher Scientific, Waltham, MA, USA) from total RNA purified from *N. benthamiana* leaves with Plant Total RNA purification Mini Kit (Favorgen, Ping-Tung, Taiwan). Due to the large size of NbImp7, amplification from *N. benthamiana* cDNA was performed in two fragments using primers 3328/3276 and 3275/3247. Fragments were cloned into pENTRY plasmid (Invitrogen, Waltham, MA, USA) digested with *Bstx*I and *Eco*RV using the Gibson assembly kit (New England BioLabs, Ipswich, MA, USA). All the clones that we obtained presented a STOP codon mutation in the middle of the sequence, suggesting this protein was deleterious for the bacteria. To overcome this problem, one of the plasmids with the STOP codon (pENTRY-Imp7^STOP^) was used as a template for the amplification of two PCR products using primers 2940/3351 and 3352/3353. Fragments were joined together by overlapping PCR using primers 2940/3353, and the product was digested with *Sph*I and *Sac*I and cloned into pUC19 plasmid digested with the same enzymes (pUC-Imp7Frag). This new plasmid was used as a template for two PCR reactions using primers 705/3367 and 3368/706. Another PCR was performed using as a template pPPV with the primers 3366/3369. All three PCR products were joined together by overlapping PCR using primers 705/706, and the PCR product was digested with *Sph*I and *Sac*I and cloned into the original pENTRY-Imp7^STOP^ digested with the same enzymes. The plasmids pBIFP2-Imp7, pGWB702Ω-Imp7, pGWB702Ω-SIIIP1b, and pBIFP3-P1 were constructed using the GATEWAY system. pBIFP2-Imp7 and pGWB702Ω-Imp7 were obtained by LR recombination of pENTRY-Imp7 with pBIFP2 [47] and pGWB702Ω [48], respectively. pDONR207-SIIIP1b was obtained by BP recombination of pDONR207 (Invitrogen, Waltham, MA, USA) with an SIIIP1b fragment amplified by PCR using as a template pPPV-SIIIP1b and primers 3558/890. LR recombination of this plasmid with pGWB702Ω [48] yielded pGWB702Ω-SIIIP1b. pDONR207-P1 was obtained by BP recombination of pDONR207 with a P1 fragment amplified by PCR using as a template pPPV and primers 3818/3099. LR recombination of this plasmid with pBIFP3 [47] yielded pBIFP3-P1. pBIFP3-P1b, pBIFP3-P1b_C89A_ and pBIFP3-P1b_RK68,69AA_ have been previously reported [35], as has mRFP-NLS [49]. pBIN61:p19 and pGFP were kindly provided by Prof. David Baulcombe (University of Cambridge, Cambridge, UK).

### 2.2. Agroinfiltration

Plants of *N. benthamiana* wild type and of the GFP transgenic line 16C [50] were grown in a greenhouse maintained in a 16 h light/8 h dark cycle and a temperature range of 19–23 °C. Plants were infiltrated as described [34], using cultures of the *Agrobacterium tumefaciens* strain C58C1-313 [51], carrying the indicated binary plasmid at an OD_600_ of 0.5 in all cases except for the bacteria carrying pGWB702Ω-Imp7 or the corresponding empty plasmids that were used at an OD_600_ of 0.75.

### 2.3. SIIIP1b Purification

Purification was performed following the reported protocol [52]. Briefly, tissue from 6 plants of *N. benthamiana* inoculated with either pPPV-SIIIP1b or pPPV, collected at 12 dpa, was smashed under liquid nitrogen and resuspended in a binding buffer (25 mM Tris-HCl, pH 8.0; 550 mM NaCl, 5 mM NaF, 0.5 mM EDTA, 10% glycerol (*v*/*v*), 0.1 mM PMSF). Samples were centrifuged, and the supernatant was collected. After adding avidin to a final concentration of 100 µg/mL, the sample was mixed with Strep-Tactin resin (IBA, Göttingen, Germany), previously cross-linked with BS3 (Thermo Fisher Scientific, Waltham, MA, USA), and left incubating for 30 min at 4 °C in a spin column (Corning, New York, NY, USA). Three cycles of centrifugation and wash (25 mM Tris-HCl, pH 8.0, 500 mM NaCl, 5 mM NaF, 0.4 mM EDTA, 0.2% Igepal CA-360 (*v*/*v*), 5% glycerol (*v*/*v*), 0.1 mM PMSF) were performed before elution of the bound proteins with the corresponding buffer (25 mM Tris-HCl, pH 8.0, SDS 1% (*w*/*v*)).

### 2.4. Mass Spectrometry Analyses

pPPV- and pPPV-SIIIP1b-eluted samples were applied to a conventional SDS-PAGE gel and stopped as soon as the front entered the resolving gel. Unseparated protein bands were excised and processed automatically in a Proteineer DP (Bruker Daltonics, Bremen, Germany). Digestion protocol was reported [53]. Half of each digested sample was subjected to 1D-nano LC ESI-MSMS analysis using a nano liquid chromatography system (Eksigent Technologies nanoLC Ultra 1D plus, SCIEX, Foster City, CA, USA) coupled to a high-speed Triple TOF 5600 mass spectrometer (SCIEX, Foster City, CA, USA) with a Nanospray III source. The analytical column used was the silica-based reversed phase Acquity UPLC^®^ M-Class Peptide BEH C18 Column (Waters, Milford, MA, USA). The trap column was a C18 Acclaim PepMapTM 100 (Thermo Fisher Scientific, Waltham, MA, USA) switched online with the analytical column. Peptides were separated using a 40 min gradient ranging from (98% A–2% B) to (10% A–90% B) (A: 2% acetonitrile, 0.1% formic acid; B: 100% acetonitrile, 0.1% formic acid). Data were acquired with TripleTOF 5600 System (SCIEX, Foster City, CA, USA), using an ionspray voltage floating (ISVF) set at 2300 V, curtain gas (CUR) set to 35, an interface heater temperature (IHT) of 150, ion source gas 1 (GS1) set at 25, and declustering potential (DP) set at 100 V. All data were obtained using information-dependent acquisition (IDA) mode with Analyst TF 1.7 software (SCIEX, Foster City, CA, USA). Mass spectrometry data obtained were processed using PeakView v2.2 Software (SCIEX, Foster City, CA, USA) and searched using Mascot Server v2.5.0 (Matrix Science, London, UK) against *N. benthamiana* protein database from Solgenomics (last update: 10 July 2015, 57.153 sequences), together with viral sequences and commonly occurring contaminants.

### 2.5. Image Analyses

BiFC images were acquired at 6 dpa by confocal laser scanning microscopy using a Leica TCS SP8 system with laser line WLL2 and HyD detectors. YFP detection was done using a laser line at 514 nm (16.7%) and HyD at 520 and 558 nm. RFP detection was done using a laser line at 560 nm (33.5%) and HyD at 575 and 628 nm. For GFP fluorescence images taken with a Leica MZFLIII epifluorescence microscope, we used excitation and barrier filters at 470/40 and 525/50 nm. Images were acquired with an Olympus DP70 digital camera at the indicated times.

### 2.6. Fluorescence Analysis

GFP fluorescence intensity quantification was carried out placing individual 5.0 mm diameter leaf discs collected at 5 dpa in a black 96-well plate (Nunc) filled with 100 µL water/well and acquiring the signal in a monochromator-based plate reader (Infinite M200, Tecan Group, Männedorf, Switzerland) [54]. Two discs per plant were collected. Statistical analysis was performed by Student’s *t*-test (*p* < 0.01).

### 2.7. RT-PCR and -qPCR

Total RNA was extracted from *N. benthamiana* plants using Plant Total RNA Purification Mini Kit from Favorgen (Ping-Tung, Taiwan) following manufacturer´s instructions. cDNA was prepared using Superscript III (Thermo Fisher Scientific, Waltham, MA, USA). SIIIP1b amplification for checking the presence of the N-terminal tag was performed using primers 90/1444. qPCR reactions were prepared with technical triplicates using HOT FIREPol EvaGreen qPCR Mix Plus (Solis BioDyne, Tartu, Estonia) in 96-well optical plates and run in a 7500HT Fast Real-Time PCR system (Applied Biosystems, Waltham, MA, USA). Primer pairs for NbImp7 (3242/3243) were selected using Primer3 [55]. Primers for GFP amplification (3227/3228) were reported [8], as were primers for the reference gene Fbox (2808/2809) [56]. Relative quantification was performed as described [57].

## 3. Results

### 3.1. P1b Interacts with and Importin-β-Like Protein of Nicotiana benthamiana during Viral Infection

**Figure 1:**
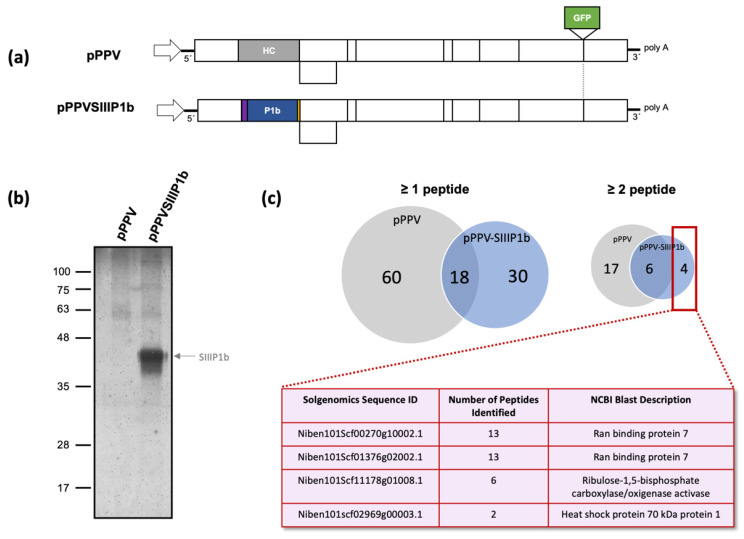
Purification and mass spectrometry analysis of SIIIP1b from PPV- and PPV-SIIIP1b-infected tissue. (**a**) Schematic representation of the two viral constructs employed in this assay. Both encode the GFP sequence between the NIb and CP regions. The purple rectangle represents the SIII tag, and the yellow rectangle represents the NIa cleavage site. (**b**) Silver staining of the SDS-PAGE analysis of PPV and PPV-SIIIP1b samples after elution from the Streptactin resin. The molecular weight is on the left; the arrow on the right indicates the estimated size of the SIIIP1b product. (**c**) Venn diagrams of the mass spectrometry results obtained from the analyzed samples using one or more peptide or two or more peptide identification. The four proteins shown in the table below correspond to the targets found exclusively in the pPPV-SIIIP1b sample.

Using a Strep tag II in tandem (SIII) at the amino terminus of an RSS gave interesting results in the case of HCPro of PVA, allowing the identification of various host protein interactors relevant for the viral life cycle [41]. Employing a similar approach, we engineered a PPV construct in which HCPro was replaced by CVYV P1b with an SIII N-terminal tag (pPPV-SIIIP1b) to facilitate purification of the protein during viral infection (Figure 1a). A similar construct expressing P1b without a tag was previously tested, showing that the chimeric virus was functional [58,59]. We inoculated by agroinfiltration six plants with pPPV-SIIIP1b and six plants with pPPV, which expresses the native HCPro, as a negative control. Both viruses express GFP as an engineered reporter of viral infection. Upper non-inoculated leaves of infected plants showing strong GFP fluorescence signal were collected at 12 days post agroinfiltration (dpa). The presence of the SIII tag and the intact P1b sequence in the genome of the viral progeny was confirmed by RT-PCR before SIIIP1b purification (data not shown). Following the protocol described [52], we purified SIIIP1b from the pPPV-SIIIP1b-inoculated tissue and performed in parallel the same purification process using the tissue inoculated with pPPV (Figure 1b). The final eluted samples were analyzed by mass spectrometry, producing a list of peptides for each sample matching the proteins of *N*. *benthamiana*. Seventy-eight host proteins were identified in the pPPV sample, and forty-eight were retrieved in the case of pPPV-SIIIP1b (Table S1). Most of the proteins were identified by the presence of a single matching peptide. When at least two peptides were included as a requisite for identification, the list was reduced to 23 proteins for the pPPV sample and 10 proteins for pPPV-SIIIP1b. From those, six proteins were common to both samples and there were only four proteins unique in the case of the SIIIP1b containing sample (Figure 1c). Two of these proteins represented two alleles of the same gene, an importin-β-like protein similar to Importin 7 of *A. thaliana* (ran binding protein 7, also termed SAD2). This protein will be hereinafter referred to as NbImp7. The other two proteins were an activase of RuBisCO and a heat shock protein. The experiment was repeated, and the results obtained were similar, with the identification of NbImp7 and the other two proteins as the primary targets of the pPPV-SIIIP1b-infected sample (Table S1).

### 3.2. Interaction between NbImp7 and P1b Occurs in the Nucleus of the Cell, Depends on a Correct Folding of P1b, but Is Independent of Its Ability to Bind siRNA

**Figure 2:**
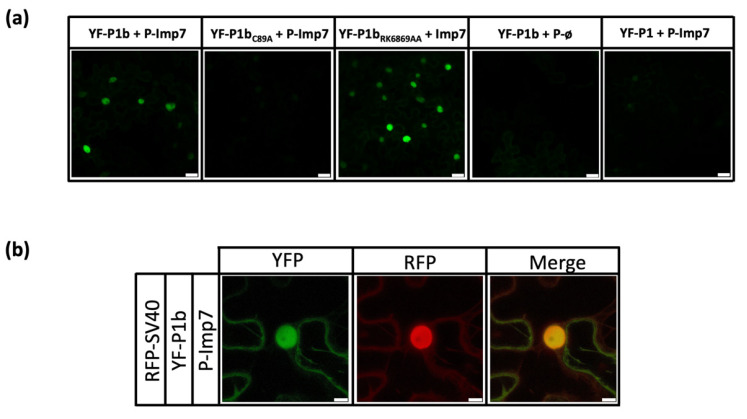
BiFC analysis to test the interaction of P1b with NbImp7 and its cellular localization. Images were taken with a confocal microscope at 6 dpa. (**a**) P1b and two P1b mutants, as well as P1, were expressed together with P-Imp7 and were tested for YFP fluorescence complementation. An empty plasmid, P-ø, was used as the negative control. The white bar indicates 25 µM. (**b**) YFP, RFP, and merge fluorescence of the same sample agroinfiltrated with the indicated constructs. The white bar indicates 10 µM.

To confirm an already identified interaction between a viral protein and a host protein and discard false positives due to technical issues, it is important to test the interaction by an alternative method [60]. We decided to use bimolecular fluorescence complementation assays (BiFC) for this, since it allowed us to evaluate the interaction, testing at the same time its intracellular location and providing a good opportunity to check different P1b mutants. NbImp7 was cloned by Gybson assembly after amplification of the gene by RT-PCR from *N. benthamiana* tissue. The protein appeared to be toxic for bacteria, and final cloning was not possible until an intron was introduced in the middle of the NbImp7 coding sequence. NbImp7 was cloned into the plasmid pBIFP2 to express the C-terminal part of YFP at its N-terminus (P-Imp7), and the rest of the viral proteins tested were cloned into the plasmid pBIFP3 to express the N-terminal part of YFP also at the N-terminus (YF-protein of interest). BiFC experiments were carried out twice using two independent clones of each construct and agroinfiltrating two leaves from two different plants. To avoid RNA silencing, a plasmid expressing tombusviral RSS P19 was included in all cases. Samples were analyzed under confocal microscopy at 6 dpa. Obtained results were consistent between constructs and between experiments. Initial BiFC assays showed a green fluorescence signal when YF-P1b was co-infiltrated with P-Imp7 while showing no fluorescence when YF-P1b was co-expressed with an empty plasmid P-ø (Figure 2a). P1b_C89A_ mutant, a protein that displays an aberrant structural conformation [35], was used as a negative control for the NbImp7 interaction, and, as anticipated, it showed no fluorescence complementation with P-Imp7. These results confirmed the previously observed interaction between P1b and NbImp7 in *N. benthamiana*. However, P1b_RK68,69AA_, a double mutant that preserves the standard P1b structure but lacks siRNA binding ability [35], showed strong fluorescence, indicating that siRNA binding is not relevant for the NbImp7-P1b interaction.

Due to the described relationship between the NbImp7 protein of *A. thaliana* (SAD2) and two potyviral P1 proteins [61,62], we also performed BiFC analysis using PPV P1 fused to YF (YF-P1). The result showed that, at least under these experimental conditions, there is no interaction between these two proteins (Figure 2a). Focusing on the cellular location of the complementation, it appeared that the interaction between NbImp7 and P1b was taking place in the nucleus of the cell. To verify this, a plasmid carrying a nuclear protein from SV40 marked with an RFP tag at the N-terminus (mRFP-NLS) [49] was co-agroinfiltrated with YF-P1b and P-Imp7. The merged results confirmed the initial observation, showing that the YFP fluorescence and the RFP nuclear fluorescence were coincidental (Figure 2b).

### 3.3. NbImp7 Is Overexpressed in the Plant after P1b Expression and during a Viral Infection

**Figure 3:**
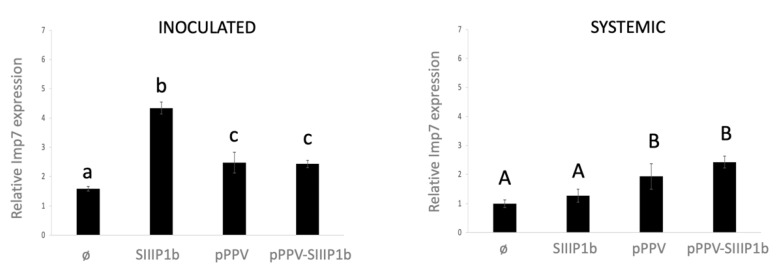
Evaluation of NbImp7 mRNA levels after P1b transient expression or after viral infection. RT-qPCR experiments to assess the mRNA expression of NbImp7, taking as reference the value of this mRNA in upper leaves of mock inoculated plants. Samples of inoculated leaves (**left**) were collected at 6 dpa; samples of systemically infected leaves (**right**) were collected at 12 dpa. Analysis of variance (*n* = 4; *p* < 0.05) (one-way ANOVA), followed by Tukey´s post hoc test, was performed; and letters indicate the different groups.

It is not uncommon for host proteins involved in a viral infection to suffer some rearrangements on their expression either by up- or by downregulation, during the pathogen attack [63]. To understand a little better the behavior of NbImp7 after potyviral infection and more specifically during P1b overexpression, we tested the mRNA levels of NbImp7 of the corresponding tissue by quantitative PCR (qPCR). We agroinfiltrated four *N. benthamiana* plants (two clones per construct and two plants per clone) with either an empty plasmid, a plasmid expressing SIIIP1b, or the viral plasmids pPPV and pPPV-SIIIP1b. Inoculated leaves were collected at 6 dpa for analysis, while upper non-inoculated leaves were collected at 10 dpa, after verifying the presence of GFP fluorescence in plants agroinfiltrated with the viral constructs. Analysis of the qPCR data (Figure 3) revealed strong upregulation of NbImp7 mRNA levels upon SIIIP1b overexpression in the inoculated leaves (left panel) compared to the control, while preserving similar NbImp7 mRNA levels in the upper non-inoculated leaves (right panel). However, a significant increase compared to the control, although not as pronounced as in the case of SIIIP1b overexpression, was also observed in the case of pPPV and pPPV-SIIIP1b infection, in inoculated and upper systemic leaves (left panel and right panel, respectively).

### 3.4. Expression of NbImp7 Interferes with P1b Ability to Suppress RNA Silencing

**Figure 4:**
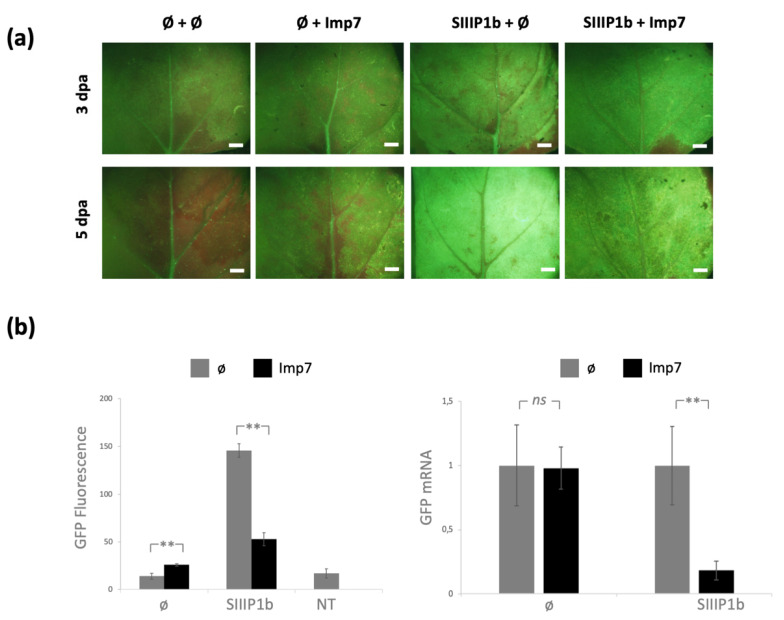
Effect of NbImp7 overexpression on SIIIP1b RNA silencing suppression activity. (**a**) Microscope images from 16C line *N. benthamiana* plants agroinfiltrated with the couple of indicated plasmids and pGFP, taken at 3 dpa and 5 dpa. The white bar indicates 2 mm. (**b**) Quantitative analyses of GFP expression of agroinfiltrated leaves collected at 5 dpa. To the left, GFP fluorescence relative to the signal obtained from wild type *N. benthamiana* plants; NT, non-treated 16C line *N. benthamiana*. To the right, RT-qPCR analysis of GFP mRNA expression relative to control plants agroinfiltrated with pGFP and only an empty plasmid. Statistical analysis by *t*-test was performed (*ns* = not significant; ** = *p* < 0.01).

Considering that NbImp7 mRNA in *N. benthamiana* plants was upregulated upon P1b expression (Figure 3), it was reasonable to assume that an artificial overexpression of NbImp7 could have an effect, positive or negative, on this RSS, providing clues as to what could be the role of this protein during viral infection. Thus, a transient co-expression system was employed and to better assess the RNA silencing capacity of SIIIP1b, 16C line *N. benthamiana* plants expressing GFP were used. Four plants per condition (two plants per clone) were agroinfiltrated in each case. Empty vectors pGWB702Ω and SIIIP1b were tested, with and without NbImp7 co-expression. Plants were co-agroinfiltrated with a plasmid encoding GFP (pGFP), and they were monitored at 3 dpa and 5 dpa (Figure 4a), collecting samples at 5 dpa. GFP fluorescence was measured by taking discs of the corresponding leaves, and the same discs were used for RNA extraction and qPCR analysis (Figure 4b). In the case of SIIIP1b samples, GFP fluorescence showed a clear downregulation when NbImp7 was co-expressed with the suppressor instead of the empty plasmid (left panel). This result was coincidental with the qPCR analysis (right panel), in which a significant reduction in the mRNA levels of GFP could be observed when NbImp7 was included in the mix. When pGWB702Ω was tested instead of SIIIP1b, the effect of NbImp7 in GFP fluorescence was the opposite. However, the qPCR results did not show variation in GFP mRNA levels whether NbImp7 was co-agroinfiltrated or not.

## 4. Discussion

Type B P1 proteins are one of the two main RSSs employed by viruses of the *Potyviridae* family to counteract the RNA silencing defense mechanism of the plant. Their mode of action as an RSS is well described, binding siRNAs [35,36] and large RNAs [37] or directly interfering with AGO1 protein [64]. Nonetheless, knowledge about the host factors they interact with during a viral infection is scarce [40,64].

We decided to study type B P1 protein CVYV P1b and find possible interactors in *N. benthamiana* plants. A chimeric virus expressing this RSS instead of HCPro was used, and an N-terminal SIII tag was employed to help in the identification process. Analysis of the P1b-associated samples by mass spectrometry revealed the presence of an interesting candidate, clearly identified in two independent experiments: an importin-β-like protein, homolog to Importin 7 protein of *A. thaliana* (Figure 1). Interaction was further confirmed by BiFC assays, which also showed that correct folding, but not the siRNA binding capacity of P1b, was relevant for the interaction (Figure 2a). The nuclear localization of the protein interplay (Figure 2b) was confirmed using a nuclear marker protein tagged with RFP. Transport to the nucleus of viral RSSs is not unusual [65,66,67] and in some cases is mandatory for their activity as suppressors [68]. However, the mode of action of P1b, sequestering siRNAs, suggests that the cytoplasm should be its primary location. It is possible that P1b is developing an unknown function in the nucleus, but it is also possible that the protein in that compartment is actually trapped in an undesirable location. Considering that Importin 7 of *A. thaliana* was described to help in the transport to the nucleus of transcription factor MYB4 [42], it is reasonable to speculate that NbImp7 could be doing the same with P1b in *N. benthamiana* plants.

It is interesting to note that type A P1 proteins are described to travel to the nucleus during viral infection [69] and appear to have Importin-7-like proteins as possible interactors [61,62]. We tested by BiFC the possible relationship between type A P1 of PPV and NbImp7, but the result was negative (Figure 2a). This was not strange considering the different viruses (PPV vs. PSbMV and TuMV) and the different experimental hosts (*N. benthamiana* vs. *Pisum sativum* and *A. thaliana*) used. Nonetheless, it seems clear that this importin is in a close relationship with P1 viral proteins, whether type A or type B, and this could suggest that the conserved protease domain was involved in the interaction.

The large upregulation of NbImp7 transcripts upon SIIIP1b expression reinforces the relationship of these two proteins and suggests that their interaction could be functional (Figure 3). The high expression of NbImp7 during pPPV-SIIIP1b infection goes along this line and could be related to the expression of the SIIIP1b RSS in the plant. Observing the same effect on NbImp7 expression during pPPV infection, which encodes no P1b, adds complexity to the picture. It is possible that PPV P1 is involved in this behavior, but the BiFC results (Figure 2a) suggest otherwise. Two other possibilities are considered: (i) the viral infection per se causes the upregulation of NbImp7 mRNA, and (ii) HCPro is playing the role of SIIIP1b and causes NbImp7 upregulation. The latter possibility would suggest that NbImp7 is able to interact also with the structurally unrelated HCPro. Although uncommon, a promiscuous behavior of this type has already been described for the endogenous RSS rgsCaM [70], which interacted with different kinds of viral RSSs, such as potyviral HCPro, cucumoviral 2b, and even with the Tat protein of human immunodeficiency virus. Further investigation is needed to elucidate this issue.

The transient expression experiments performed with SIIIP1b and NbImp7 (Figure 4) shed some light on the effect that this host protein overexpression could be having during viral infection. Interestingly, the expression of NbImp7 alone had a positive effect on GFP fluorescence, indicating an increase in protein accumulation. This increase could be due to an RNA silencing suppression activity of NbImp7, given that a similar function was already described for its *Arabidopsis* homolog [44]. If this was the case, and considering that the difference in fluorescence was not accompanied by a difference in mRNA accumulation, NbImp7 would not be acting by suppressing mRNA degradation but by relieving silencing derived from translational repression [71,72]. However, the results obtained with SIIIP1b were solid, indicating a strong reduction both at the protein and at the mRNA levels. Overall, this last result suggests that NbImp7 might be hampering the role of SIIIP1b as an RSS either by hijacking the protein in the nuclear compartment, as previously suggested, or by other means, such as proteasome-mediated degradation, as was the case of rgsCaM [70], siRNA modulation/sequestration, etc. Thus, the interaction between SIIIP1b and NbImp7 could be involved in the plant antiviral defense mechanism. Nonetheless, considering some precedents [73,74], we cannot discard the possibility that the virus was using this repression to modulate its own RNA silencing suppression activity, favoring viral infection.

Identifying new interactions between host factors and viral proteins is a difficult but necessary task, since it is this interplay that reveals the actual functions and mechanisms behind the viral infection. We identified a new interaction between an importin-β-like protein, NbImp7, and a type B P1 protein, P1b. Our data suggest this interaction is functional, affecting viral RSS behavior inside the cell. Understanding the role of this interplay will improve our knowledge about potyviral infections and could help define a new function of NbImp7 in the defensive system of the plant, as well as reveal unknown functions of the potyviral RSSs in the nucleus of the cell. Future research will help verify the relevance of this interaction and its involvement during viral infection.

## Data Availability

The data that support the findings of this study are available from the corresponding author upon reasonable request.

## Supplementary Materials

The following are available online at https://www.mdpi.com/article/10.3390/v13122406/s1, Table S1: Mass spectrometry analyses, Table S2: List of primers used in the study.
