# Supplementary material for: An Importin-β-like Protein from Nicotiana benthamiana Interacts with the RNA Silencing Suppressor P1b of the Cucumber Vein Yellowing Virus, Modulating Its Activity
Source: Viruses. 2021 Nov 30;13(12):2406. doi: 10.3390/v13122406 (PMC8706682; doi:10.3390/v13122406)
Supplement: Supplementary file 1 [file viruses-13-02406-s001.zip › viruses-1481963-supplementary/TableS2.pdf]

Table S2. List of primers used in the study

Name	Sequence 5´-> 3´
2712	TTGGTCTCATCCACAATTCGAGAAGGGAGG
2711	GCATAAAAGTCAATTTTATCTTTC
3328	GTACAAAAAAGCAGGCTTTAAAGGAACCATGGATCTTCAGAACCTTG
3276	CTGAACAAGCATACGCTCAAGCTCTGATTTGTAAGGTTCA
3275	TGAACCTTACAAATCAGAGCTTGAGCGTATGCTTGTTCAG
3247	CTTTGTACAAGAAAGCTGGGTCTAGATCTAAGCAGCTGTTGCTGATG
2940	GCAAAGCATTACACAGTGTTGTGG
3351	CCAAAGGCTTTGTTGGTAGTC
3352	GACTACCAACAAAGCCTTTGG
3353	CGTTGCAACAAATTGATAAGC
705	CGACGTTGTAAAACGACGGCC
3367	GGTAGAAGCAGAAACTTACCTGCCCCTTGCAGTGC
3368	CCAAAATTTGTTGATATGCAGGTGGATCACTGGGTTG
706	CGGATAACAATTTCACACAGG
3366	GCACTGCAAGGGGCAGGTAAGTTTCTGCTTCTACC
3369	CAACCCAGTGATCCACCTGCATATCAACAAATTTTGG
3558	GGGGACAAGTTTGTACAAAAAAGCAGGCTCCATGTCTGATTGGTCTCATCCACAATTC
890	GGGGACCACTTTGTACAAGAAAGCTGGGTCACAATAAAAGTCAATTTTATCTTTCTCATCTGC
3818	GGGGACAAGTTTGTACAAAAAAGCAGGCTTCATGTCAACCATTGTATTTGGCTCATTCAC
3099	GGGGACCACTTTGTACAAGAAAGCTGGGTTAGTAGTGGATTATCTCATTGC
90	CGGACCCAATGCAAG
1444	GACTGCAGGGTGCACATC
3242	TTGGAGATTCCGAAGCAGTT
3243	CCAAACCGGGTGTATAGTCG
3227	CAACTTCAAGACCCGCCACA
3228	TCTGGTAAAAGGACAGGGCCA
2808	GGCACTCACAAACGTCTATTTC
2809	ACCTGGGAGGCATCCTGCTTAT
